# Supplementary material for: Knee joint distraction in regular care for treatment of knee osteoarthritis: A comparison with clinical trial data
Source: PLoS One. 2020 Jan 22;15(1):e0227975. doi: 10.1371/journal.pone.0227975 (PMC6975543; doi:10.1371/journal.pone.0227975)
Supplement: Attachment — Submitted filename: ResponseToReviewers.pdf [file pone.0227975.s002.pdf]

We thank the reviewer for the helpful comments by which we consider the manuscript improved. Hopefully we answered all questions and addressed all comments to the intention and satisfaction of the reviewer.

- Review comment:

  Remove the name of your hospital UMC from all pages of the article. You can write: our hospital instead.

- Author response:

  As suggested, we removed the hospital name 'UMC Utrecht'. We either replaced it with 'our hospital' [lines 28, 74, 164, 197, 399] or completely removed it [line 398] when appropriate. However, we did not remove 'University Medical Center Utrecht' when referring to the ethical review committee in the methods section [line 98/99] because this refers not to the hospital but to the medical ethical review committee that approved the studies (as required to mention according to the PLOS ONE submission guidelines).

- Reviewer comment:

  In line 97, remove the country name Netherland and hospital name....remove all the Line as no need.

- Author response:

  As suggested, we removed any reference to The Netherlands ('Dutch') in [line 24, 66, 398] and replaced 'Dutch' with 'local' in [line 75, 377]. However, we did not remove 'Netherlands Trial Register' in the methods section [line 99/100] because this is the official name of where the trials were registered and can be found online (as required to mention according to the PLOS ONE submission guidelines).

- Reviewer comment:

  In table 2, what do you mean by Corpus liberum? and why breaking of bone pin happen?

- Author response:

  The corpus liberum was a loose piece of cartilage/bone present in the knee. This short explanation has been added in [line 246]. It is unfortunately not known why the bone pin broke, and as we do not want to speculate on this without being sure, we added 'reason unknown' in the complications section of the

revised manuscript [line 233].

- Reviewer comment:

  In discussion: you referred the high pin tract infection in the trial group to not taking antibiotic regularly as happened in regular practice group! But actually, there are many factors influence this! The rate of pin tract infection in publications range from 0-100%! This means that it is 0% in hands of someone who follow some tricks while it is 100% in some other hands! To improve that, a proper new drill bit with the right diameter should be chosen and proper drill speed used and a soothing saline should be used not a dry drilling technique! A proper 6 mm. Diameter pins should be used and there should not be any stress to any pin in the Ex Fix. As any stress will be reflected to the bone and leads to loosening abs infection.

- Author response:

  We agree that there are many factors that affect the amount of pin tract infections. As mentioned in the results section [line 228], a new wound care protocol already managed to decrease the amount of pin tract infections from 85% in the first clinical trial (the open prospective study) to 57% in the following clinical trials (the randomized controlled trials). In the regular care group the percentage of antibiotic use was with 70% a bit higher than in the RCTs and we hypothesize that this increased percentage may be because the infections are not diagnosed in person by a physician, but patients always receive a standard prescription of oral antibiotics to take in case of suspected pin tract infections. As such, the amount of patients taking antibiotics is not necessarily the same as the amount of patients experiencing pin tract infections. Nevertheless, we should aim to decrease the amount of pin tract infections and antibiotics courses as much as possible by indeed making appropriate changes to the regular care protocol, which we are doing by using the newly developed distraction device and evaluating new regular care protocols, as mentioned in the discussion [line 348-351]. We will also take your suggestions into account in future adjustments to hopefully decrease the amount of pin tract infections.

- Reviewer comment:

  In line 308, you pointed to a new device used in the study: ArthroSave. Did you use in all patients? Please add a figure for a photo of the new device to the study.

- Author response:

  The mention of the company name ‘ArthroSave’ was removed in the previous revised version of the manuscript on request of the other reviewers, in order to remove any potential conflict of interest regarding the new device. This newer device (the KneeReviver) was not used on any of the patients used in this manuscript and is currently being analyzed in an ongoing clinical study. We mention it in the discussion only because this new device is specifically designed to decrease pin tract infections, as these infections increase the patient’s treatment burden. However, as none of the patients in this study were treated with this device, we did not add a photo of this new device to avoid confusion.

- Reviewer comment:

  Please add a brief surgical technique to the study as it is valuable for the reader to know how to do KJD in the future.

- Author response:

  We added some extra information regarding the surgical technique in the revised version of the manuscript [line 105-116].

- Reviewer comment:

  The X Ray in figure 1, does not show clearly the planes of pins! Are they pure medial and lateral? Kindly describe.

- Author response:

  The planes of the pins can indeed not be accurately visualized in the radiograph in figure 1. The pins are not positioned purely medially and laterally and an explanation of how the pins should be positioned was included in a brief description of the surgical technique. We added some extra information regarding the surgical technique in the revised version of the manuscript [line 105-116].

- Reviewer comment:

  I can see 2 arthrosave devices were applied in the sane fig. Please explain why 2 devices and not one is enough?

- Author response:

  One distraction device consists of two separate tubes that are both fixed to the femur and tibia with bone pins, one laterally and one medially, as highlighted in the methods sections [line 105-106]. The distraction treatment is always performed on both sides of the knee, and as such with two tubes, so that both the medial and lateral side are distracted properly. A unilateral frame with one tube provides insufficient distraction to the contralateral compartment because of a certain degree of flexibility in the distraction frame and pins.

- Reviewer comment:

  In the same fig. I can see proximal clamp on the medial side showing a mechanical problem of the pins as pins are divergent. The distance between the 2 pins are closer at the clamp level while it is longer at the bone level. It is clear that the distal pin is blended. Actually this leads to a big stress to the bone cortex and can leads to many complications as pin tract infection, loosening, pin breakage or bone breaking as you mentioned 2 cases with this problem among the trial group which is about 5%! This is really much and if the pins were inserted properly without any stress then the complication rate will be much less. I would like if you can choose another X Ray showing the device with distraction with a proper technique as many young specialists colleagues will see your fig. In the future and try to copy so we must provide them with the right technique. I am sure that you can choose better X Ray to show.

- Author response:

  We agree that pin positioning is very important and the goal is to position the pins perpendicularly instead of divergent. It is therefore indeed better to choose a radiograph with perpendicular and parallel positioning and replaced figure 1 with an image where the pins are positioned fully correct.

- Reviewer comment:

  What are the pin diameter you used? Please write this in the study as it is important to know. Also write it for the regular care group.

- Author response:

  We agree that this is a detail that should be included. As mentioned we extended the brief explanation on the used surgical technique [line 105-116] and in this part we also included the pin diameter (5mm). As mentioned in the methods section of the manuscript [line 142] the treatment procedure was identical for the trial group and regular care group.

- Reviewer comment:

  I can see a long tapered tip of the pins that are not introduced well to pass the far cortex of the bone. This could be a direct cause of instability and loosening and infection. Please explain that or remove this fig and choose a better one with the proper technique to give a good example to the reader.

- Author response:

  It is indeed important that the pins properly pass the far cortex of the bone, as described in the extended surgical technique description in the revised methods section [line 105-117]. However, the radiograph is performed in AP direction and the pins of especially the tibia are positioned under an angle and not straightly in medial-lateral direction, which means the pins protrude to the second cortex of the bone slightly on the posterior side. Because of this, the AP radiograph do not show the pins exit the bone, even though they do.
