# Supplementary material for: Knee joint distraction in regular care for treatment of knee osteoarthritis: A comparison with clinical trial data
Source: PLoS One. 2020 Jan 22;15(1):e0227975. doi: 10.1371/journal.pone.0227975 (PMC6975543; doi:10.1371/journal.pone.0227975)
Supplement: Attachment — Submitted filename: ResponseToReviewers - Final.docx [file pone.0227975.s001.docx]

We thank the reviewers for the helpful comments by which we consider the manuscript improved. Hopefully we answered all questions and addressed all comments to the intention and satisfaction of the reviewers.

Reviewer 1

- Review comment:
  My comment is to do more thorough discussion about complications, not only infections, how did you treat, how these complications had influenced the outcome, etc.
- Author response:
  We agree that it is relevant to know how all complications were treated in regular care, not just focusing on the pin tract infections being the main complication. Therefore, in the revised Results section [line 220-235], we now mention for all types of complications how they were treated. While in the Results and the Discussion section we already included that experiencing complications did not have a significant influence on clinical benefit, we made this more explicit with the addition of the relevant p-value in the Results section [line 279-280]. Furthermore, we included a paragraph in the revised Discussion section [line 340-348] about the influence of complications other than pin tract infections on the clinical response. Complications other than pin tract infections did not occur in sufficient numbers to statistically test the influence of each complication on the clinical response, but we used the patients’ clinical experience as mentioned during their outpatient clinic visits after treatment to be able to elaborate on the potential effect of the complications on the clinical benefit.
- Review comment:
  Another comment is to add more figures to the paper to show the effect of distraction.
- Author response:
  We appreciate the comment, however, standardized radiographs or MRIs are not part of the protocol in in regular care, and as such are not available. This means we cannot objectively measure the structural effect of knee joint distraction in patients treated in regular care. In the three clinical trials we have previously demonstrated significant effect such as an increase in radiographic joint space width or improved cartilage thickness on MRI. We agree that the (structural) effect of distraction is interesting, and therefore added two examples (representatives of clear radiographic jointspace widening) of a trial and regular care patient [figure 2].
  However, since data on structural repair are not available for regular care patients in quantitative measures and this outcome has already been published for trial patients, we feel that a focus on patient characteristics, complications and clinical effect should be the key message of the present manuscript. We referred to the added radiographs and included this point of discussion in the revised Discussion section of the manuscript [line 364-373].
- Reviewer comment:
  Third comment is about other radiographic tools, MRI, CT, etc, was there any place for such modalities in diagnosis or follow up of your cases?
- Author response:
  Other radiographic tools, especially MRI, would indeed provide useful information in these patients. Unfortunately, no quantitative radiographic tools other than a non-standardized radiograph at inclusion demonstrating a Kellgren-Lawrence grade of at at least 2 (standard inclusion characteristic in the Dutch guidelines for knee joint replacement indication) and non-standardized radiographs post-treatement were used, because of the cost and the absence to use them for indication according to guidelines. Specifically for indication in future studies such imaging tools may be of help in regulare care to include the optimal population for this treatment, which is why we mention this shortly in the Discussion [line 402-403].

Reviewer 2:

- Reviewer comment:
  The article presents no control group to objectively assess the outcome.
- Author response:
  The goal of this research was to compare patients treated in regular care to those treated in clinical trials, the latter being the control group for the regular care group. We agree that inclusion of patients that do not receive any treatment would be on interest. However, the population considered in this study comprise patients that present themselves in the hospital with a condition of end-stage knee OA needing surgical treatment, with in regular care indication total knee prosthesis but below the age of 65 years. As such any good control group receiving no or only conservative treatment, which all patients have had according to Dutch guidelines before they can be considered for arthroplasty, would not be ethical for this population. In the clinical trials, other control groups have previously been used: participants from the osteoarthritis initiative matched on patient characteristics with the KJD patients (Van der Woude et al, *Cartilage* 2017), and in the two randomized controlled trials patients treated with total knee arthroplasty (Van der Woude et al, *Bone Joint J* 2017; Jansen et al, *Cartilage* 2019) or high tibial osteotomy (Van der Woude et al, *Knee* 2017; Jansen et al, *Cartilage* 2019). Since such a comparion is not the key message of the present manuscript and these comparisons have been published already, we did not include them in this present manuscript. This choice has been added in the discussion section of the revised manuscript [line 290-394].
- Reviewer comment:
  There is risk of bias from the conflict of interest, the authors used a device they gain financial benefit from (ArthroSave BV) .
- Author response:
  This study is an investigator-initiated study and no industrial funding is involved, nor involvement in any orther way. Moreover, all patients in the trials and in regular care were treated with a proof of concept device and not with ArthroSave’s KneeReviver. ArthroSave’s KneeReviver was introduced less than a year ago, so there is still no sufficient follow-up. To avoid confusion, we removed reference to the company name (ArthroSave BV) from the revised Discussion section when referring to the new dedicated user-friendly frame provided by ArthroSave with limited occurrence of pin tract infections [line 337].
  A risk of bias that we did recognize is the relatively high percentage of regular care patients that did not fill out both baseline and one-year follow-up questionnaires, as we acknowledged in the Discussion section mentioning how we tried to minimize this bias [line 375-383].
- Reviewer comment:
  The follow-up is very short to get to a conclusion that this device or this technique helps to delay TKR in OA knee
- Author response:
  We agree that we cannot yet draw a conclusion about whether or not KJD treatment can postpone TKR in regular care after this one year of follow-up. However, all trials have demonstrated the long-term clinical and structural benefit obtained (e.g. Jansen et al OAC 2018 Dec;26(12):1604-1608). The maximum (optimum) effects in all these studies was around 1 year, sustaining for years thereafter. As such we feel that this one-year follow-up comparison is indicative for long-term follow-up as well. However as this needs confirmation in regular care we rephrased the final sentence of the revised manuscript [line 405-409].
